# Comparison of pulse wave analysis parameters by oscillometry in hypertensive diabetic and nondiabetic patients in a Brazilian outpatient care

**DOI:** 10.1097/MD.0000000000018100

**Published:** 2019-12-16

**Authors:** Luiz Antonio Pertili Rodrigues Resende, Marco Antonio Vieira Silva, José Augusto Mantovani Resende, Elisabete Aparecida Mantovani Rodrigues Resende, Valdo José Dias Silva, Dalmo Correia

**Affiliations:** a Department of Internal Medicine; b Department of Tropical Medicine and Infectiology, Federal University of the Triângulo Mineiro, Uberaba, Minas Gerais, Brazil.

**Keywords:** arterial stiffness, diabetes, hypertension, oscillometry, pulse wave analysis

## Abstract

**Introduction:**

Pulse wave analysis is an emerging approach that analyzes parameters comprising strong predictors of cardiovascular (CV) events and all-cause mortality, especially in patients with high CV risk based on established risk factors. This study used the oscillometric method, provided by the Mobil-o-Graph (PWA-EMI GmbH, Stolberg, Germany) device, to compare data regarding the pulse wave analysis parameters in hypertensive nondiabetic and diabetic patients.

**Material and methods:**

In this cross-sectional study, 276 individuals were examined in the academic hypertension outpatient care unit of the Federal University of the Triângulo, in Mineiro, Brazil, from January to December 2016. The pulse wave analysis was performed by oscillometry, and its parameters were acquired from all patients.

**Results:**

Of the 276 patients, 99 were diabetic and 177 nondiabetic. The mean systolic and pulse central blood pressure were significantly higher in diabetic patients than in nondiabetic patients (*P* = .008 and.0003, respectively). The mean peripheral systolic blood pressure and pulse pressure were also significantly higher in the diabetic group (*P* = .001 and *P* < .0001, respectively). The average pulse wave velocity (PWV, m/s) was 9.4 ± 1.6 and 8.8 ± 1.6 in the diabetic and nondiabetic groups, respectively (*P* = .003).

**Conclusion:**

The group of hypertensive diabetic patients had significantly higher central blood pressure, peripheral blood pressure, and PWV than the hypertensive nondiabetic patients. The patients with overlapping established CV risk factors presented values of the pulse wave analysis parameters consistent with higher central pressure and greater arterial stiffness.

## 1 Introduction

Cardiovascular (CV) disease is a leading cause of morbidity and mortality worldwide, and its features are not entirely predicted by classic risk factors and diagnostic approaches. Increasing importance has been attributed to arterial components, namely, endothelial dysfunction and arterial stiffness.^[1]^ Metabolic alterations in the endothelium that are associated with the inflammatory state, aging process, and atherosclerosis lead to the diminished arterial elasticity and compliance.^[2,3]^ Consequently, the evaluation and measurement of arterial stiffness comprise a promising approach in predicting, staging, and managing CV risk.^[4–6]^

The most reliable parameter in determining arterial stiffness is pulse wave velocity (PWV), which is the major component of pulse wave analysis. A pulse wave is generated at each systole, and its components depend on left ventricular function and arterial characteristics. Thus, the analysis of the pulse wave by a reliable, noninvasive, reproducible, and inexpensive technique constitutes a useful method in predicting CV risk and mitigating CV events.^[7,8]^

Recently, a new oscillometric method that is noninvasive and reproducible has been developed. This method allows the measurement of peripheral and central blood pressure and pulse wave analysis parameters, notably the PWV, through the transformation of the brachial pulse waveform.^[9]^ This new approach to access pulse wave parameters is feasible for outpatient use and therefore is a simple and useful equipment for primary care units. Validation studies suggest a strong correlation with invasive intra-aortic catheter measurements, which is the gold standard.^[10–12]^

Currently, the aortic PWV is a known strong predictor of CV events and all cause mortality, especially in patients who already have high CV risk based on established risk factors.

Hypertension is a pathologic condition with concerning prevalence worldwide.

The increase in blood pressure levels has a wide range of effects in the body, and the arteries are frequently involved. Hypertension is already considered an important cause of arterial stiffness and is related to high PWV values and other components of pulse wave analysis such as the augmentation index (AugI) and augmentation pressure (AugP).^[13]^

Diabetes is also a morbid condition regarding its effects to the arteries. Individuals with diabetes have greater PWV compared with the general population even when adjusted for age, blood pressure, and heart rate. Diabetes is also described as an independent marker of CV disease in patients with diabetes.^[14,15]^

Hypertension is an important and powerful modifiable risk factor of CV diseases in patients with type 2 diabetes. The rate of hypertension among diabetic individuals is up to 75%, which points to an important association regarding CV physiopathology alterations in these 2 conditions. This suggests an additive effect in the arterial stiffness process, evaluated mainly by the PWV. The purpose of this study was to evaluate the importance of diabetes and hypertension, as well as its coexistence, in the behavior of arterial pulse wave analysis parameters^[14,16,17]^ by comparing hypertensive patients with and without diabetes.

## 2 Materials and methods

### 2.1 Study population

This prospective cross-sectional study included 276 hypertensive individuals undergoing regular follow-up at the academic hypertension outpatient care unit of the Federal University of Triângulo Mineiro (FUTM) and Diagnostic Cardiac Center (DCC), located in Uberaba, Minas Gerais, Brazil. Informed consent was obtained and documented. The patients were examined from January to December 2016. Patients with hypertension and diabetes who were attending regular follow-up at our outpatient care were included. Patients younger than 18 years, with underlying condition(s) that required recent hospitalization, heart failure functional class 3 or 4 of the New York Heat Association, and end-stage kidney disease were excluded. The protocol of the study followed the principles of the Declaration of Helsinki and was approved by the institutional ethics committee. Written informed consent was obtained from all participants.

### 2.2 Pulse wave analysis and clinical data

After a rest period of at least 30 minutes, the oscillometric method of pulse wave analysis was performed in an appropriate room, between 9:00 AM and 3:00 PM, during peak period of antihypertensive medication. The device used, Mobil-O-Graph (PWA-EMI GmbH, Stolberg, Germany), is a commercially available brachial oscillometric ambulatory blood pressure monitor that has been validated according to the recommendations of the European Society of Hypertension^[12,18,19]^ and also registers the peripheral and central blood pressure levels. A common cuff was centered to the left upper arm. Cuff size was chosen according to the circumference of the mid upper arm. Generation of central aortic blood pressure curves based on brachial pulse waves is based on a previously published algorithm that integrates arterial impedance and aortic hemodynamics into a mathematical model. After digitalization, the signal processing is performed using a 3-level algorithm. In a first step, the single pressure waves are verified for their plausibility by testing the position of minima and the corresponding wavelengths. During the second stage, all single pressure waves are compared with each other to recognize artifacts. Thereafter, an aortic pulse wave is generated by the means of a generalized transfer function. The idea behind a transfer function is the modification of a certain frequency range within the acquired pulse signal to get the aortic pressure wave.^[11,20]^

Four consecutive measurements were taken every 2 minutes according to general recommendations for clinical studies.^[8]^

All individuals answered questions about personal and clinical data (medication use, CV risk factors, and personal and family history of CV disease). Patients also underwent conventional sphygmomanometer arterial blood pressure measurement according to the guidelines of the Brazilian Hypertension Society. The diagnosis of hypertension was based on the same guidelines.^[21]^

The hypertensive diabetic patients were diagnosed according to the American Diabetes Association recommendations^[22]^ and were attending regular follow-up at the diabetes outpatient care of the FUTM. We did not select any specific subgroup of diabetic patients.

### 2.3 Statistical analysis

We built a database using the Excel program and performed the statistical analysis using the GraphPad Prism (San Diego, CA) and MedCalc software. All results are expressed as mean ± standard deviation. Chi-squared test was applied to compare proportions and Student *t* test signed rank test continuous variables. Given unequal sample sizes of our data, multivariate analyses (ANOVA) with D’Agostino–Pearson test was used to compare pulse wave analysis parameters and performed adjustment for age, smoking, mean antihypertensive agents per patient, mean body mass index, mean abdominal circumference (cm). For PWV adjustment we add peripheral systolic blood pressure (pSBP), peripheral mean blood pressure (pMBP), peripheral pulse pressure (pPP), central systolic blood pressure (cSBP), central pulse pressure (cPP), and AugP.

## 3 Results

### 3.1 Study population

Among the 276 hypertensive patients, 177 were nondiabetic and 99 were diabetic. There were 173 women (62.6%), and the average age of the patients in the nondiabetic and diabetic groups was 58 **±** 11.9 and 59 ± 11 years, respectively. A total of 272 (98.5%) patients were taking antihypertensive medication. The population characteristics are given in Table 1.

**Table 1:**
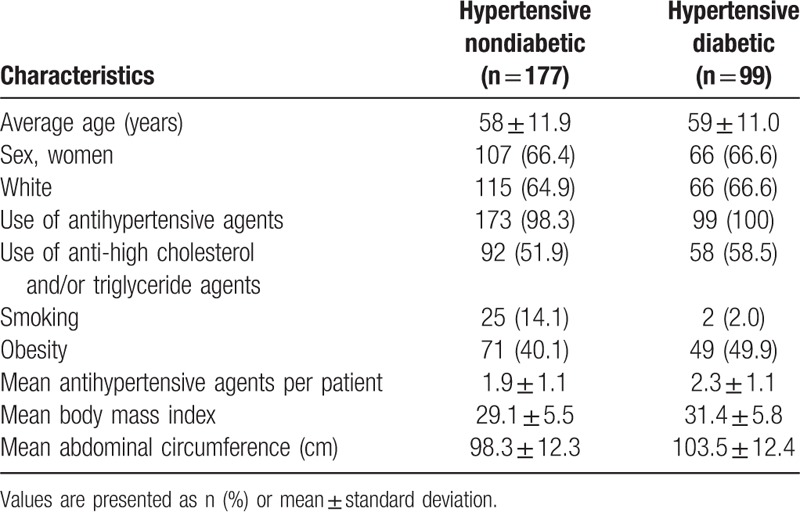
Study population.

### 3.2 Comparison of pulse wave analysis parameters

The mean cSBP and pulse pressure were significantly higher in the diabetic group in comparison with the nondiabetic group (*P* = .008 and .0003, respectively) (Table 2). We obtained the same difference in the comparison of the peripheral blood pressure: the mean systolic and pulse pressures were also significantly higher in the diabetic group (*P* = .001 and *P* < .0001, respectively). The average PWV was 9.4 ± 1.6 and 8.8 ± 1.6 in the diabetic and nondiabetic groups, respectively (*P* = .003).

**Table 2:**
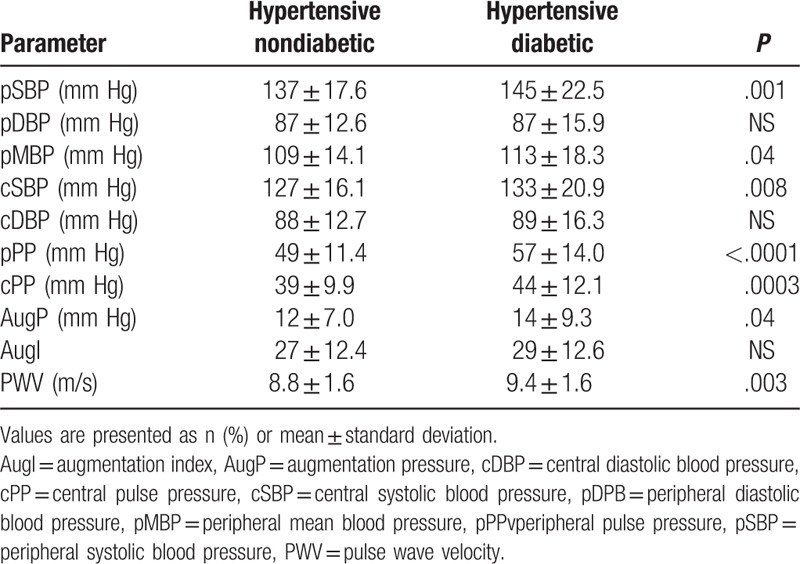
Pulse wave analysis among diabetic and nondiabetic hypertensive patients.

After adjustment for diabetic vs nondiabetic comparisons, these differences remained significant for the following variables: PWV 9.1 ± 0.05 vs 8.9 ± 0.03 (*P* = .002), pSBP 143 ± 1.9 vs 138 ± 1.4 (*P* = .0003), pPP 56 ± 1.3 vs 50 ± 0.9 (*P* = .0001), cPP 42 ± 1.1 vs 39 ± 0.9 (*P* = .01). And missed significant values for: pMBP 112 ± 1.6 vs 110 ± 1.2 (*P* = .38), cSBP 131 ± 1.8 vs 128 ± 1.3 (*P* = .18), and AugP 14 ± 0.8 vs 12 ± 0.6 (*P* = .10).

**Figure 1:**
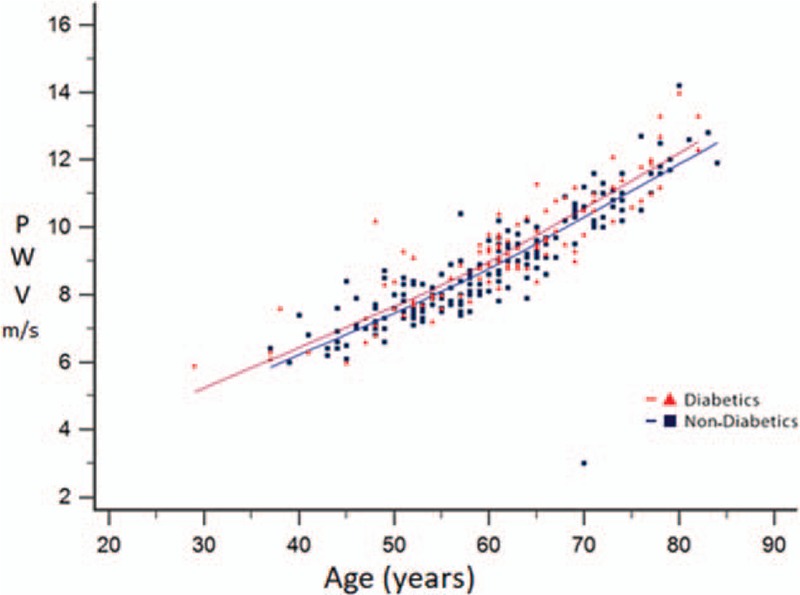
Correlation between age and pulse wave velocity among 276 hypertensive nondiabetic and diabetic patient. PWV = pulse wave velocity.

The PWV values of the hypertensive diabetic patients were more elevated regardless age variation (Fig. 1).

The AugP was also higher in patients with diabetes (*P* = .04). There was no statistical difference between the average AugI and the diastolic central or peripheral blood pressure (cDBP; pDBP).

## 4 Discussion

The oscillometric method of pulse wave analysis is a new noninvasive approach to determine arterial stiffness. The data are collected easily and without any discomfort to the patient, and the results are equivalent to those of invasive methods.^[9–12]^

The results of the present study suggest the positive association regarding pulse wave analysis parameters and the known risk factors for CV outcomes such as hypertension and diabetes. The peripheral Blood pressure, central and peripheral pulse pressures were significantly difference between the groups, which illustrates the relevance of the overlapping of risk factors in the genesis of morbidity related to CV diseases. The results were predictable and concordant with the current knowledge on this topic.^[13–16,23–26]^

Diabetes and hypertension represent 2 extremely important conditions when considering global health due to the substantial prevalence and outcomes involved. These conditions are associated with relevant arterial impairment. There are many mechanisms involved in the genesis of arterial stiffness in hypertensive and diabetic patients. Besides mechanical injury, there is an inflammatory aspect that is not fully understood. Modern medicine demands new approaches on how to best evaluate these patients using simple techniques that are applicable to large populations.^[3,27]^

In a review based on data obtained from the French cohort, DESIR (Data from an Epidemiologic Study), diabetic patients (n = 126) showed significant higher PWV values (11. 5 vs 13.9; *P* = .0001) than nondiabetic patients (n = 203).^[15]^ Compared with our study, the patients on DESIR had higher systolic blood pressure values (158 ± 29 vs 160 ± 28) than our study (137 ± 17.6 vs 145 ± 22.5). Surely the differences in PWV between the studies at least in part could be explained by a difference in blood pressure.^[28]^ Using a new method to measure arterial stiffness, equally, we found significant differences in arterial stiffness between diabetic and nondiabetic patients, which reinforce our results.

Our study had some limitations. The stratification of the study subjects could have been performed according to the time of diagnosis and control of the underlying disease. There was no control group of healthy individuals, which could estimate the real impact of hypertension and diabetes in the pulse wave parameters. In the diabetic group there was a higher prevalence of smoking patients; however, we could not find data about the association between the smoking habit and the PWV. An important aspect of the sample analysis was that the PWV was higher in the hypertensive diabetic patients regardless of age variation. Age is a factor that presents an independent association with increased arterial stiffness and could be a confounding variable in the analysis.^[3,4]^

The development and application of technologies that are simple, routine, cheap, and relevant are great value, especially in developing countries like Brazil where CV and metabolic diseases are important public health problems. In this context, the establishment of approaches that allow the early identification of risk factors become essential in the promotion of health in its primary level of care and prevention.^[21]^

Furthermore, our study raised the need for approaches that take into account the diagnostic time of diabetic patients and their glycemic control. The drug used in the treatment of hypertension is also a relevant aspect to be considered. The comparison between different glycemic levels and the CV repercussion on pulse wave parameter should motivate more studies.

In conclusion, in this study, hypertensive diabetic patients had significantly higher central and peripheral systolic pressure and pulse pressure compared with hypertensive nondiabetic patients. The AugP was also statistically higher in hypertensive diabetic patients. Furthermore, diabetic patients had higher PWV regardless of age variation, and the difference was statistically significant. This study demonstrated that patients with overlapping established risk factors for CV disease had altered pulse wave analysis parameters consistent with arterial stiffness and central hemodynamic disorders.

## Author contributions

**Conceptualization:** Luiz Antonio Pertili Rodrigues Resende, Marco Antonio Vieira Silva, Jose Augusto Mantovani Resende, Elisabete Aparecida Mantovani Rodrigues Resende, Valdo Jose Dias Silva, Dalmo Correia.

**Data curation:** Luiz Antonio Pertili Rodrigues Resende, Marco Antonio Vieira Silva, Jose Augusto Mantovani Resende, Dalmo Correia.

**Formal analysis:** Luiz Antonio Pertili Rodrigues Resende, Marco Antonio Vieira Silva, Jose Augusto Mantovani Resende.

**Investigation:** Luiz Antonio Pertili Rodrigues Resende, Marco Antonio Vieira Silva, Jose Augusto Mantovani Resende, Dalmo Correia.

**Methodology:** Luiz Antonio Pertili Rodrigues Resende, Marco Antonio Vieira Silva, Jose Augusto Mantovani Resende, Valdo Jose Dias Silva, Dalmo Correia.

**Project administration:** Luiz Antonio Pertili Rodrigues Resende, Marco Antonio Vieira Silva, Jose Augusto Mantovani Resende, Dalmo Correia.

**Resources:** Luiz Antonio Pertili Rodrigues Resende, Marco Antonio Vieira Silva, Jose Augusto Mantovani Resende, Dalmo Correia.

**Software:** Luiz Antonio Pertili Rodrigues Resende, Marco Antonio Vieira Silva, Jose Augusto Mantovani Resende, Dalmo Correia.

**Supervision:** Luiz Antonio Pertili Rodrigues Resende, Marco Antonio Vieira Silva, Jose Augusto Mantovani Resende, Dalmo Correia.

**Validation:** Luiz Antonio Pertili Rodrigues Resende, Marco Antonio Vieira Silva, Jose Augusto Mantovani Resende, Dalmo Correia.

**Visualization:** Luiz Antonio Pertili Rodrigues Resende, Marco Antonio Vieira Silva, Jose Augusto Mantovani Resende, Dalmo Correia.

**Writing - Original Draft:** Luiz Antonio Pertili Rodrigues Resende, Marco Antonio Vieira Silva, Jose Augusto Mantovani Resende, Elisabete Aparecida Mantovani Rodrigues Resende, Valdo Jose Dias Silva, Dalmo Correia.

**Writing - Review & Editing:** Luiz Antonio Pertili Rodrigues Resende, Marco Antonio Vieira Silva, Jose Augusto Mantovani Resende, Elisabete Aparecida Mantovani Rodrigues Resende, Valdo Jose Dias Silva, Dalmo Correia.
